# Patterns, predictors and subsequent outcomes of disease progression in metastatic renal cell carcinoma patients treated with nivolumab

**DOI:** 10.1186/s40425-018-0425-8

**Published:** 2018-10-17

**Authors:** Haris Zahoor, Pedro C. Barata, Xuefei Jia, Allison Martin, Kimberly D. Allman, Laura S. Wood, Timothy D. Gilligan, Petros Grivas, Moshe C. Ornstein, Jorge A. Garcia, Brian I. Rini

**Affiliations:** 1 Taussig Cancer Institute, Cleveland Clinic, Cleveland, OH USA; 2 Department of Quantitative Health Sciences, Lerner Research Institute, Cleveland Clinic, Cleveland, OH USA; 3 Department of Hematology and Oncology, Taussig Cancer Institute, Cleveland Clinic, Lerner College of Medicine, 9500 Euclid Avenue, Cleveland, OH 44195 USA

**Keywords:** Renal cell carcinoma, Clear cell, Immunotherapy, Nivolumab, Biomarker, Failure

## Abstract

**Background:**

Nivolumab is approved for the treatment of refractory metastatic renal cell carcinoma. Patterns and predictors of progressive disease (PD) on nivolumab, and outcomes in such patients are lacking.

**Methods:**

A retrospective analysis of patients (pts) with metastatic clear cell renal cell carcinoma (ccRCC) who received nivolumab at Cleveland Clinic (2015–2017) was performed. PD was defined per Response Evaluation Criteria in Solid Tumors (RECIST) v1.1 or clinical progression as per treating physician. Univariate analyses (UVA) and multivariate analyses (MVA) were used to identify clinical and laboratory markers as potential predictors of progression-free survival (PFS).

**Results:**

Ninety patients with mean age of 65, 74% men, and 83% good or intermediate International Metastatic Renal Cell Carcinoma Database Consortium (IMDC) risk group were included. Median number of prior systemic treatments was 2 (range, 1–6). Median overall survival (OS) and PFS were 15.8 and 4.4 months, respectively. Fifty-seven patients (63%) had PD and 44% of patients with radiographic PD had new organ sites of metastases with brain (8/23, 35%) being the most common. Twelve patients received treatment beyond progression (TBP), and among 6 patients with available data, 3 (50%) had any tumor shrinkage (2 pts. with 17% shrinkage, one pt. with 29% shrinkage). Of 57 patients with PD, 28 patients (49%) were able to initiate subsequent treatment, mainly with axitinib and cabozantinib, while 40% of patients were transitioned to hospice after PD. In MVA, a higher baseline Neutrophil-to-Lymphocyte ratio (NLR) (HR, 1.86; 95% CI, 1.05–3.29; *p* = 0.033) was associated with an increased risk of progression, whereas higher (> 0.1 k/uL) baseline eosinophil count was associated with a lower risk of progression (HR, 0.54; 95% CI, 0.30–0.98; *p* = 0.042).

**Conclusion:**

Brain was the most common site of PD in patients treated with nivolumab, and only half of patients progressing on nivolumab were able to initiate subsequent treatment. The risk of PD increased with a higher baseline NLR and reduced with a higher baseline eosinophil count.

**Electronic supplementary material:**

The online version of this article (10.1186/s40425-018-0425-8) contains supplementary material, which is available to authorized users.

## Background

The treatment of advanced clear cell renal cell carcinoma (ccRCC) has dramatically changed over the last decade with introduction of targeted agents including tyrosine kinase inhibitors (TKI) [1]. Although these agents have significantly improved outcomes, they rarely result in complete responses [2, 3].

Renal cell carcinoma has been considered an immune-responsive tumor and immunotherapy with high dose IL-2 has been used in select patients leading to complete and durable responses in a subset of patients [4]. More refined and novel immunotherapies have been developed due to improved understanding of T cell function and associated immunosuppressive molecules such as cytotoxic T-lymphocyte-associated protein 4 (CTLA-4), program death 1 (PD-1) and PD-1 ligand 1 (PD-L1), called immune checkpoints [5].

Nivolumab, a fully human IgG4 anti-PD- antibody, is the first approved checkpoint inhibitor for the treatment of metastatic RCC refractory to antiangiogenic therapy based on a phase III clinical trial [6]. As compared to everolimus, a mammalian target of rapamycin (mTOR) inhibitor, nivolumab improved overall survival (OS) (HR: 0.73 *p* = 0.002). The overall response rate with nivolumab was 25% vs. 5% with everolimus (*p* < 0.001). The treatment was well tolerated with 19% treatment related grade 3 or 4 Adverse Events (AEs) in nivolumab vs. 37% in everolimus patients. Based on these data, nivolumab became the preferred standard of care treatment for metastatic RCC patients who have progressed on previous antiangiogenic therapy.

Although nivolumab has prompted a paradigm shift in the treatment of metastatic RCC, only a subset of patients benefit from this treatment, and hence identifying predictive biomarkers is an area of active research. The CheckMate 025 trial investigated the role of PD-L1 expression as a marker of response [6]. Patients with higher PD-L1 expression were shown to have worse outcomes as compared to those with low PD-L1 expression. However, both groups appeared to derive the same benefit from nivolumab relative to everolimus, indicating that PD-L1 expression was prognostic but not predictive and thus cannot be used to select patients for treatment. Similarly, little is known about the patterns of disease progression and outcomes of patients who progress on nivolumab treatment. The main objective of this analysis was to evaluate patterns and predictors of failure, and subsequent outcomes in patients treated with nivolumab. These data can generate hypotheses regarding markers of response to select appropriate patients for treatment, and also provide prognostic information to patients and physicians.

## Methods

After obtaining approval from Institutional Review Board of Cleveland Clinic, we performed a retrospective review of patients with advanced ccRCC who received nivolumab at Cleveland Clinic (2015–2017). Data on patient characteristics, treatment patterns and clinical follow up was extracted from chart review. Baseline laboratory parameters at the time of treatment of initiation, including Neutrophil-to-Lymphocyte ratio (NLR), absolute eosinophil count, absolute monocyte count and absolute basophil count, were also extracted from chart review.

Patients were divided into two groups at a three-month landmark. The first group, called the PD group, was comprised of patients with progressive disease as their final outcome at the time of analysis. The second group, called NPD, was comprised of patients who had not progressed on nivolumab at time of analysis. PD was defined per Response Evaluation Criteria in Solid Tumors (RECIST) v1.1 or clinical PD defined as lack of clinical benefit from nivolumab as per treating physician discretion. The interval of radiographic response evaluation was not predefined although generally done every 12 weeks and baseline neuroimaging was not routinely done.

### Statistical analyses

Categorical clinic-pathologic factors were summarized. A landmark analysis at 3 months was performed to explore any potential differences in baseline characteristics between PD and NPD groups. Fisher’s exact text and the Wilcoxon rank sum test were used to compare clinic-pathologic factors between two groups. OS and PFS were summarized using the Kaplan-Meier method. PFS was defined as the time from the first dose of nivolumab to radiographic or clinical progression or death, whichever came first, censored at last follow-up for patients who had not progressed. OS was calculated as the time from the first dose of nivolumab to the date of death or last follow-up. Cox proportional hazards models were used for comparisons between factors. A *p* value ≤0.05 was regarded as significant. Univariate analyses (UVA) were used for clinic-pathologic factors and baseline patient characteristics. The multivariable analysis (MVA) was performed by using the step-wise variable selection with IMDC and adjusted for number of prior treatment and prior treatment with IL-2 or interferon (IFN) (Additional file 1), and was used to identify potential predictors of progression-free survival (PFS). Recursive partitioning method was used to identify cut-off values for NLR and eosinophil counts. All data analyses were carried out using R software (3.5.0).

## Results

### Baseline patient characteristics

Ninety patients with mean age of 65 (SD, 9.88) were included in the analysis. Of these, 74% were men and 82% had Eastern Cooperative Oncology Group (ECOG) Performance Status of 1–2. Eighty-three percent of patients had a good or intermediate International Metastatic Renal Cell Carcinoma Database Consortium (IMDC) risk category [7]. The median number of prior systemic treatments was 2 (range, 1–6). Prior nephrectomy was done in 97% of patients. Sunitinib (71%) was the most common prior treatment used. (Table 1).

**Table 1:** Baseline Patient Characteristics

Characteristics	No (%)*n* = 90
Mean age, years (SD)	65 (9.88)
Male Gender	67 (74)
ECOG PS	
0	34 (41)
1	33 (40)
> 2	15 (18)
IMDC Risk Group	
Favorable	12 (14)
Intermediate	61 (69)
Poor	15 (17)
Prior Nephrectomy	67 (97)
No of prior systemic therapies, median, No. (range)	2 (1, 6)
No of prior systemic therapies	
1	42 (47)
2	24 (27)
3	16 (18)
4	6 (7)
> 5	2 (2)
Most common prior systemic therapies	
Sunitinib	64 (71)
Pazopanib	30 (33)
Axitinib	35 (39)
Sites of metastases at baseline	
Brain	14 (16)
Bones	37 (41)
Lungs	65 (72)
Liver	27 (30)
Lymph Nodes	58 (64)
Pleural	18 (20)
Adrenal	20 (22)

The baseline characteristics of patients in the PD and NPD groups at 3 months after initiating nivolumab were similar except higher incidence of baseline lung (85% vs. 63%, *p* = 0.046), lymph node (79% vs. 53%, *p* = 0.019) and pleural metastases (33% vs. 10%, *p* = 0.016) in PD group. (Table 2).

**Table 2:** Comparison of PD and NPD using landmark analysis at 3 months

Characteristics	PD Group N (%) *n* = 49	NPD Group N (%) *n* = 39	*p*-value
Mean age, years (SD)	66 (10.20)	64 (9.61)	0.401
Male Gender	33 (67)	33 (85)	0.107
ECOG PS			0.106
0	23 (52)	10 (27)	
1	15 (34)	18 (49)	
> 2	6 (14)	9 (24)	
IMDC Risk Group			0.139
Favorable	8 (17)	4 (10)	
Intermediate	35 (73)	24 (63)	
Poor	5 (10)	10 (26)	
Prior Nephrectomy	35 (97)	30 (97)	1.000
No of prior systemic therapies, median, No. (range)			
No of prior systemic therapies			0.404
1	25 (51)	15 (38)	
2	10 (20)	14 (36)	
3	10 (20)	6 (15)	
> 4	3 (6)	4 (10)	
Common prior systemic therapies			
Sunitinib	38 (78)	24(61)	0.161
Pazopanib	15 (31)	15 (38)	0.586
Axitinib	18 (37)	17 (44)	0.665
Sites of metastases at baseline			
Brain	7 (18)	7 (14)	0.862
Bones	13 (33)	24 (49)	0.208
Lungs	33 (85)	31 (63)	0.046
Liver	14 (36)	12 (24)	0.352
Lymph Nodes	31 (79)	26 (53)	0.019
Pleural	13 (33)	5 (10)	0.016
Adrenal	9 (23)	11 (22)	1.000

Two patients were excluded from this analysis because of lack data regarding their PD status

Common sites of metastases at baseline included lung (72%), lymph nodes (64%) and bone (41%). Brain metastases were present in 14 (16%) patients. All patients had received central nervous system (CNS)-directed therapy (Whole brain radiation treatment; 2 patients, Gamma Knife surgery; 10 patients, and surgical resection plus Gamma Knife surgery; 2 patients). Of these 14 patients, further progression of brain metastases was observed in 3 (21%) patients while receiving nivolumab. Two out of these 3 patients were treated with nivolumab beyond progression along with palliative radiation therapy. Two out of 14 patients had overall clinical deterioration, not attributed to nivolumab, and died. The remaining 9 patients had no further evidence of progression of brain metastases on nivolumab treatment.

### Efficacy summary

**Fig. 1:**
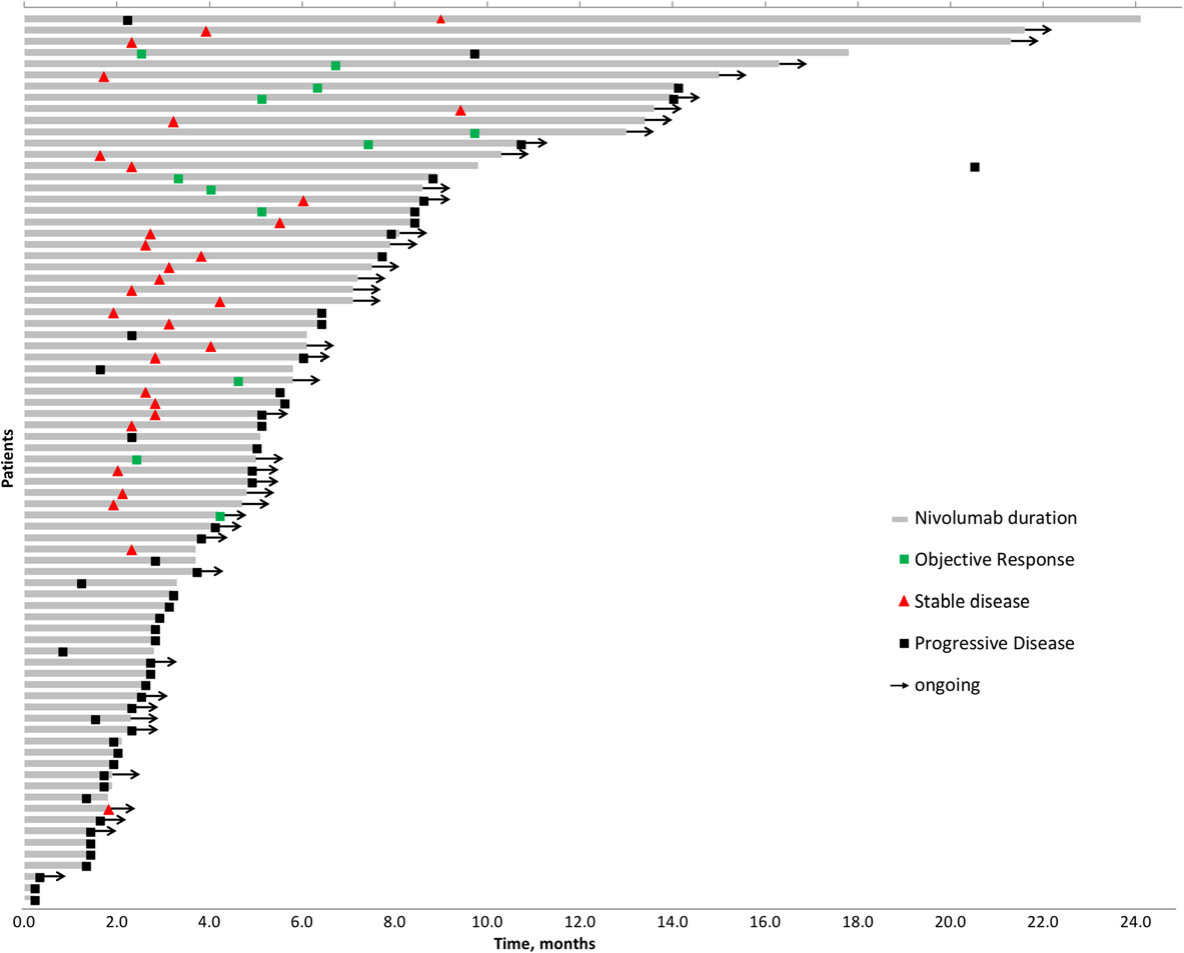
Swimmer plot of time on treatment for evaluable patients (*n* = 79)

With the median follow up of 7.6 months after initiation of nivolumab, patients remained on treatment for a median of 2.8 months. Among 79 patients evaluable for response, the overall response rate was 15% (one patient with complete response), 38% had stable disease and 47% had progressive disease as the best objective response to nivolumab. The additional 11 patients were either lost to follow up or had missing data to assess response. (Fig. 1).

The median time to response was 2.4 months. The estimated median PFS and OS were 4.8 and 15.8 months, respectively. The median PFS of patients with one prior therapy was 5 months as compared to 2.9 months in patients with more than one prior therapy (*p* = 0.54).

### Patterns of disease progression

Overall 57 patients (63%) developed PD. Among these patients, 51 (89%) had radiographic PD as per RECIST, 5 patients (9%) had evidence of clinical PD and one patient had both clinical and radiographic PD. Among patients who developed radiographic PD, 23 patients (44%) had new organ sites of metastases. The most common sites of new metastases at time PD were brain (35%) followed by liver (17%), soft tissue (17%) and loco-regional (17%). (Table 3).

**Table 3:** Patterns of disease progression and subsequent outcomes

Characteristics	n = 90
PD	57 (63)
RECIST	51 (89)
Clinical	5 (9)
Both	1 (2)
Patients with new organ sites at time of RECIST PD	23 (44)
New organ sites at time of RECIST PD	
Brain	8 (35)
Bones	1 (4)
Liver	4 (17)
Soft tissue	4 (17)
Pleural	1 (4)
Local	4 (17)
Adrenal	3 (13)
Management after PD	
Subsequent systemic treatment	28 (49)
Hospice	23 (40)
Died	3 (5)
Subsequent therapies in PD group after nivolumab discontinued	
Cabozantinib	6 (21)
Axitinib	14 (50)
Everolimus	1 (4)
Temsirolimus	3 (11)
Sunitinib	2 (7)
Others^*^	2 (7)

^*^One patient was enrolled in a clinical trial investigating an experimental drug in combination with Atezolizumab. A second patient was enrolled in a clinical trial and randomized to receive tivozanib

CNS directed local therapy was offered to all patients (3 out of 8 patients) who developed brain metastases and continued nivolumab treatment (beyond progression) in this study.

### Treatment beyond progression

Twelve patients (21%) received treatment beyond progression (TBP) with a median duration of TBP of 2.8 months (95% CI, 0.6–5.0). However, only 6 patients had follow up data available to evaluate outcomes of TBP. Among these 6 patients with available data, 3 (50%) had any tumor shrinkage. Two patients had a 17% reduction in tumor burden whereas one patient had a 29% reduction in tumor burden.

### Outcomes after disease progression

Of 57 patients with PD, 50% were able to initiate subsequent systemic treatment. Axitinib (50%) and cabozantinib (21%) were the most common subsequent treatments. Forty percent of patients were transitioned to hospice and were not able to receive any subsequent systemic treatment after progression on nivolumab. Patients who were unable to initiate subsequent treatment after progression on nivolumab appeared to be frail (ECOG PS > 2; 27% vs. 14%, *p* = 0.57) and poorer risk (IMDC poor risk 29% vs. 10%, *p* = 0.14) as compared to patients who initiated subsequent systemic treatment.

### Univariate and multivariate analyses

In univariate analysis, variables associated with poor PFS included Karnofsky performance status < 80% (HR 1.86; 95% CI, 1.03–3.35; *p* = 0.039), presence of lung metastases (HR 1.89; 95% CI, 1.04–3.41; *p* = 0.035), presence of lymph node metastases (HR 1.75; 95% CI, 1.04–2.95; *p* = 0.036), and presence of pleural metastases (HR 2.64; 95% CI, 1.47–4.74; *p* = 0.001). Baseline NLR (HR 1.03; 95% CI, 1.00–1.07; *p* = 0.05) and eosinophil count (HR 1.01; 95% CI,1.00–1.02; *p* = 0.016) were both inconclusive in univariate analysis.

In MVA, higher (> 4.2) baseline NLR (HR, 1.86; 95% CI, 1.05–3.29; *p* = 0.033) was associated with an increased risk of progression, whereas higher (> 0.1 k/uL) baseline eosinophil count was associated with lower risk of progression (HR, 0.54; 95% CI, 0.30–0.98; *p* = 0.042). Presence of baseline lung, lymph node and pleural metastases were associated with higher risk of progression in multivariate model but did not reach statistical significance. (Table 4).

**Table 4:** Multivariable analysis of PFS

Parameter	Hazard Ratio	95% Confidence Interval	p-value
Baseline Lung Metastases	1.92	0.96, 3.86	0.066
Baseline LN Metastases	1.67	0.88, 3.19	0.12
Baseline Pleural Metastases	1.69	0.86, 3.33	0.1
IMDC Intermediate Risk Group (Favorable as reference)	0.62	0.25, 1.56	0.31
IMDC Poor Risk Group (Favorable as reference)	0.51	0.16, 1.66	0.26
Baseline Neutrophil to Lymphocyte Ratio (NLR) < 4.2 vs > = 4.2	1.86	1.05, 3.29	0.033
Baseline Absolute Eosinophil Count (k/uL) < 0.1 vs > = 0.1	0.54	0.30, 0.98	0.042

## Discussion

Immune checkpoint inhibitors (ICI) have prompted a paradigm shift in many cancers including RCC [5]. Nivolumab has shown promising activity and an overall survival advantage with an excellent safety profile in refractory metastatic RCC patients. ICI are now being investigated in the first line setting either alone, or in combination with another ICI or a VEGF-directed agent. Combination treatment with nivolumab and ipilimumab, an anti-CTLA 4 antibody, was recently approved by FDA for intermediate or poor-risk RCC patients based on Checkmate 214 trial [8]. This study demonstrated a robust clinical activity of this combination, and patients had a significant lower risk of death when compared to sunitinib. Of note, in an exploratory analysis of this study involving favorable risk patients, sunitinib had improved ORR and PFS when compared to nivolumab plus ipilimumab. Similarly, a randomized phase III trial met its primary endpoint demonstrating superiority of combination of atezolizumab, an anti-PD-L1 antibody, in combination with bevacizumab, as compared to sunitinib [9]. Pembrolizumab monotherapy in treatment naïve patients has also shown promising clinical activity in a phase II trial [10]. These data suggest that ICI, either in combination with another ICI or VEGF directed agent, or monotherapy, will become standard of care for treatment naïve RCC patients in near future. Therefore, outcomes of these patients after treatment failure will be instructive to improve therapeutic options in the refractory space, and also provide prognostic information to patients and clinicians.

The present retrospective analysis demonstrated broadly similar efficacy for nivolumab monotherapy in refractory RCC as noted in the registration trial. For example, the median PFS was 4.8 months in the current analysis as compared to 4.6 months. The ORR (15% vs. 25%) and median OS (15.8 vs. 25 months) were lower than the registration trial. However, it should be noted that patients included in this analysis were more heavily pretreated when compared to the registration trial of nivolumab. Similarly, there were fewer favorable risk patients included in the current study (13% favorable IMDC risk group) as compared to the registration trial. Notably, however, a higher incidence of new brain metastases at time of PD was observed and only a subset of patients were able to initiate subsequent systemic therapy after PD.

The incidence of brain metastases in the current analysis is higher than what has been reported in RCC patients in the literature either on observation or active treatment [11]. Several hypothesis-generating explanations may explain this observation including poor permeability of the blood-brain barrier [12]. It is also plausible that higher incidence of brain metastases in current study is a reflection of natural history of disease and these events were captured more often due to a heavily pretreated and refractory patient population. Since patients with active or untreated brain metastases are often excluded from phase III clinical trials [13], level I evidence regarding the safety and efficacy of ICI in brain metastases, specifically symptomatic brain metastases, is lacking. Retrospective data suggests that metastatic RCC patients with brain metastases don’t derive benefit from nivolumab and local CNS therapy should be incorporated in the treatment plan [14].

Comparison of patients with limited benefit from ICI to those who derive more durable and substantial benefit can identify potential clinical variables, which can be used to select patients to maximize clinical benefit and avoid unnecessary toxicities. In this study, a landmark analysis at 3 months after initiating nivolumab was therefore performed to divide patients into PD and NPD groups. These two groups were then compared and showed no major differences in clinical variables. Previous studies in RCC have shown that clinical characteristics do not predict response to immunotherapy except poor IMDC risk score, which is associated with an enhanced response to treatment.

Several molecular and genetic predictive biomarkers of immunotherapy are under investigation including PD-L1 expression [15], tumor mutational burden [16], gene expression signatures [17] and tumor infiltrating lymphocytes [18]. However, reproducibility, pathologic specimen requirement, tumor heterogeneity and sampling variability, have been major challenges in the development and clinical utilization of these biomarkers [19]. Serum markers such as peripheral blood cell counts are readily available, and may predict response to immunotherapy. A higher baseline or increased absolute lymphocyte count with treatment is associated with improved response to immunotherapy and overall survival in some series [20, 21]. This association may be due to the fact that immune checkpoints are expressed on various lymphocyte populations and hence a higher lymphocyte peripheral blood count may be associated with more PD-L1 positive lymphocytes in the tumor and thus greater anti-tumor effects with immunotherapy [22, 23]. An elevated peripheral neutrophil count, on the other hand, is a marker of chronic inflammation leading to impaired immunity [24], tumor growth, metastases and poor outcomes in cancer patients [25]. In vitro studies have shown neutrophils can suppress the cytotoxic activity of lymphocytes when they are co-cultured, and this suppression is dose-dependent [26]. NLR, derived from the quotient of the absolute neutrophil count and the absolute lymphocyte count, is essentially a reflection of hemostasis between cancer inflammation and host anti-tumor response [27]. A higher NLR has been shown to be prognostic in multiple solid tumors with varying thresholds of being used to define a higher or a significant value [28]. Specifically in RCC, Templeton et al. demonstrated that RCC patients receiving targeted therapy have worse outcome with higher baseline and on-treatment increase in NLR [29]. NLR has also shown similar prognostic value in RCC patients treated with ICI [30].

An increased eosinophil count can be seen from an immuno-allergic process or lymphocytosis. Immune checkpoint inhibition can potentially lead to exacerbated allergic manifestations and animal data suggest that CTLA-4 blockade can promote allergic eosinophilic inflammation and antigen-specific IgE secretion [31, 32]. A higher baseline absolute eosinophil count or an increase in eosinophil count with treatment has been shown to correlate with improved OS in melanoma patients treated with immunotherapy [20, 21, 33]. A higher baseline eosinophil count in the current study was associated with favorable outcome, which is consistent with prior studies [20].

This study has several limitations including the selection bias of a retrospective study. Secondly, the study only included patients with clear cell histology, which limits the generalization of these findings to non-clear cell histology. Lack of independent imaging review and inconsistent intervals of response evaluation are also limitations. Clinical PD was not predefined and was based on treating physician discretion. The predictive versus prognostic value of laboratory markers evaluated in this study cannot be determined due to lack of a control arm. In addition, PFS was the clinical readout for the multivariate analysis. PFS may not be the best marker of response to immunotherapy but given lack of complete responses and variable overall survival in this heterogeneous population, it was deemed acceptable to generate a hypothesis of factors affecting outcome to nivolumab in this setting. Lastly, these patients were treated at a tertiary care academic center, which can lead to selection bias.

## Conclusions

In conclusion, this study highlights the patterns of disease progression and outcomes after disease progression in metastatic RCC patients treated with nivolumab outside of clinical trial. Further validation in larger cohorts and prospective studies is needed and may help appropriate patient selection to maximize treatment benefit and minimize toxicities.

## Additional file

Additional file 1: **Table S1:** Multivariate analysis of PFS after controlling for number of prior treatments. **Table S2:** Multivariate analysis of PFS after controlling for prior treatment with IL-2 or Interferon (DOCX 15 kb)

## Abbreviations

cc: Clear Cell
ICI: Immune checkpoint Inhibitor
PD-1: Programmed Death-1
PD-L1: Programmed Death Ligand-1
RCC: Renal cell carcinoma
IMDC: International Metastatic Renal Cell Carcinoma Database Consortium
