# Supplementary material for: Patterns, predictors and subsequent outcomes of disease progression in metastatic renal cell carcinoma patients treated with nivolumab
Source: J Immunother Cancer. 2018 Oct 17;6:107. doi: 10.1186/s40425-018-0425-8 (PMC6192175; doi:10.1186/s40425-018-0425-8)
Supplement: Supplementary file 1 — Table S1: Multivariate analysis of PFS after controlling for number of prior treatments. Table S2: Multivariate analysis of PFS after controlling for prior treatment with IL-2 or Interferon (DOCX 15 kb) [file 40425_2018_425_MOESM1_ESM.docx]

Table S1: Multivariate analysis of PFS after controlling for number of prior treatments

| **Parameter** | **Hazard Ratio** | **95% Confidence Interval** | **p-value** |
| --- | --- | --- | --- |
| Baseline Lung Metastases  Baseline Pleural Metastases  IMDC Intermediate Risk Group (Favorable as reference)  IMDC Poor Risk Group (Favorable as reference)  Baseline Neutrophil to Lymphocyte Ratio  (NLR) < 4.2 vs >= 4.2  Baseline Absolute Eosinophil Count  (k/uL) < 0.1 vs >= 0.1  No of prior treatments | 2.02  2.13  0.72  0.54  1.90  0.58  1.10 | 1.01, 4.04  1.10, 4.12  0.29, 1.75  0.16, 1.80  1.07, 3.37  0.32, 1.03  0.88, 1.37 | 0.047  0.025  0.46  0.31  ***0.028***  ***0.062***  0.40 |

Table S2: Multivariate analysis of PFS after controlling for prior treatment with IL-2 or Interferon

| **Parameter** | **Hazard Ratio** | **95% Confidence Interval** | **p-value** |
| --- | --- | --- | --- |
| Baseline Lung Metastases  Baseline LN Metastases  Baseline Pleural Metastases  IMDC Intermediate Risk Group (Favorable as reference)  IMDC Poor Risk Group (Favorable as reference)  Baseline Neutrophil to Lymphocyte Ratio  (NLR) < 4.2 vs >= 4.2  Baseline Absolute Eosinophil Count  (k/uL) < 0.1 vs >= 0.1  Prior treatment with IL-2 or Interferon | 1.92  1.67  1.69  0.62  0.51  1.86  0.54  1.00 | 0.96, 3.86  0.88, 3.19  0.86, 3.33  0.25, 1.56  0.16, 1.66  1.05, 3.30  0.30, 0.98  0.42, 2.39 | 0.066  0.12  0.13  0.31  0.26  ***0.034***  ***0.042***  0.99 |
